# Supplementary material for: Photoreactions of the C2H4–SO2 Complex in a Low-Temperature Matrix Investigated by Infrared Spectroscopy and Density Functional Theory Calculations
Source: Molecules. 2024 Nov 14;29(22):5362. doi: 10.3390/molecules29225362 (PMC11596873; doi:10.3390/molecules29225362)
Supplement: Supplementary file 1 [file molecules-29-05362-s001.zip › molecules-3311313-supplementary.pdf]

Supplementary Information

Photoreactions of the C_2H_4–SO_2 Complex in a Low-Temperature Matrix Investigated by Infrared Spectroscopy and Density Functional Theory Calculations

Taito Takahashi [1], Fumiyuki Ito [1,2,*] and Jun Miyazaki [1]

[1] Department of Natural Sciences, School of Engineering, Tokyo Denki University, 5 Senju-Asahi-cho, Adachi-ku 120-8551, Tokyo, Japan

[2] National Institute of Advanced Industrial Science and Technology (AIST), Onogawa 16-1, Tsukuba 305-8569, Ibaraki, Japan

Procedures of calculations

1. C_2H_4-SO_2 vdW complex

The initial structure of the C_2H_4-SO_2 complex was derived from the optimized structure of the C_2H_4-O_3 complex in the previous study [21]. The optimized structure of the C_2H_4-SO_2 complex at B3LYP-D_3/6-31+G(d) is compared with the R_0 structure obtained from Fourier Transform Microwave studies [14]. The distance between the centers of mass (R_{cm}) of C_2H_4 and SO_2 is calculated to be 3.360 Å; 4% underestimated compared to the experimental value of 3.504 Å. The calculation at the B3LYP-D_3/cc-pVTZ level gives R_{cm} = 3.369 Å, with a small improvement over the result with the smaller basis set.

2. Candidate molecules of UV photolytic products in the longer wavelength region (λ ~300 nm)

   69 isomers were constructed with GaussView assuming the following valence for each element; 4 for C, 2 for O, 1 for H. For sulfur atom 2, 4, 5 and 6 were assigned to take hypervalence into account. Candidate molecules with C=O bonds were eliminated due to the absence of a strong C=O stretching band around 1700-1800 cm^{-1}. After structure optimization of the 69 isomers with the semiempirical PM6 Hamiltonian, 46 isomers were found stable. All these isomers are displayed in Figure S1 and the top 14 molecules are listed in Table S1.

Figure S1. Stable isomers optimized at PM6 calculations

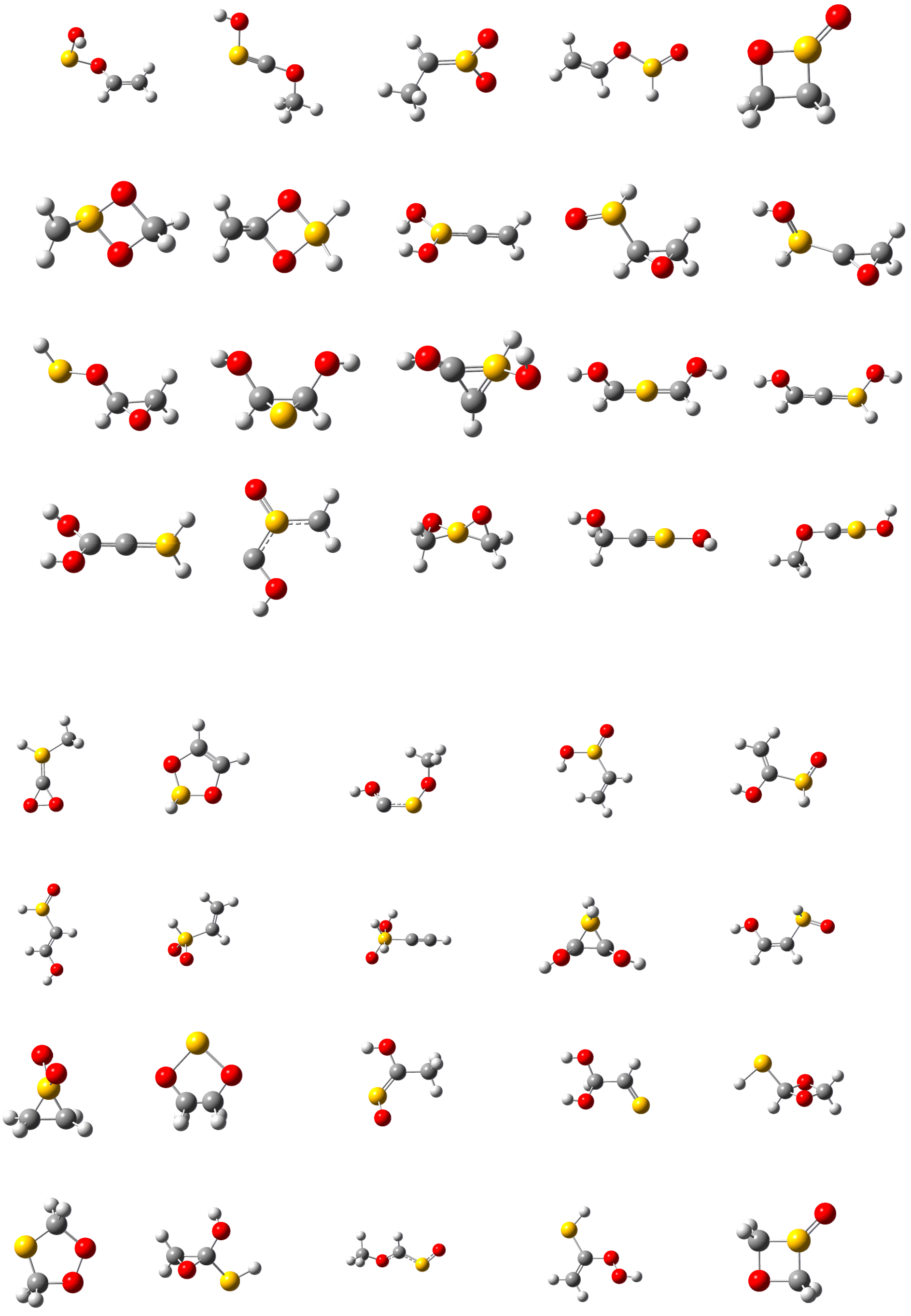

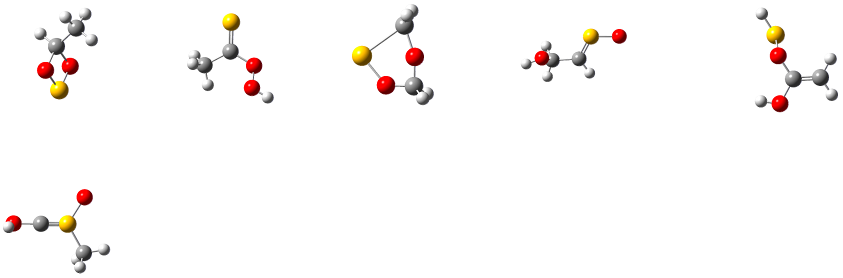

Table S1. Results of DFT calculations on the $C_2H_4SO_2$ species. [a)]

molecule	IUPAC name and index [b)]	E(a.u.) at B3LYP/6-31+G(d)	E(a.u.) at B3LYP/cc-pVTZ	ΔE(a.u.) at B3LYP/6-31+G(d)	ΔE(a.u.) at B3LYP/cc-pVTZ	Stability [c)]
$CH(OH)_2$-CHS	2,2-Dihydroxyethanethial, I	-627.2239382	-627.34553098	0.0	0	1
c-CHOH-SCHOH	II	-627.2180967	-627.34110357	0.00584152	0.00442741	2
c-CH_2-S-O-CH_2-O	1,4,2-dioxathiolane, III	-627.2095156	-627.32605982	0.01442266	0.01947116	4
CH_3-C(OH)(SO)	IV	-627.2067737	-627.33557850	0.01716457	0.00995248	3
c-CH_2-O-CH(SH)-O	1,3-dioxetane-2-thiol, V	-627.2054988	-627.32121528	0.0184394	0.0243157	5
c-CH_2-$OSOCH_2$	1,3,2-dioxathiolane, VI	-627.1920208	-627.31205486	0.03191749	0.03347612	8
c-CH_2-OS(=O)CH_2	oxathietane 2-oxide, VII	-627.1879148	-627.31447059	0.03602349	0.03106039	7
C_2H_3-S(=O)OH	ethenesulfinic acid, VIII	-627.1826604	-627.31778687	0.04127781	0.02774411	6
c-CH_2-O-CH_2-S(=O)	1,3-oxathietane 3-oxide, IX	-627.1718203	-627.29336821	0.05211798	0.05216277	10
CH_3-O^+=C^-	X	-627.1602	-627.28317	0.06364816	0.06235678	11

=S-OH		901	420			
CH_3-O-S-C^--O^+H	XI	-627.1576782	-627.28081846	0.06626001	0.06471252	13
c-CH_2-SO_2-CH_2	thiirane 1,1-dioxide, XII	-627.1470007	-627.28262764	0.07693756	0.06290334	12
CH_3-CH=SO_2	XIII	-627.1586783	-627.29851833	0.06525995	0.04701265	9
CH_3-S(=O)-$C^-$$O^+$H	XIV	-627.1208474	-627.24733807	0.10309087	0.09819291	14

[a] 14 isomers are arranged in descending order of energy obtained at B3LYP/6-31+G(d) level of theory.

[b] IUPAC name was obtained from PubChem whenever possible. An index for each molecule is a Greek numeric to represent stability at B3LYP/6-31+G(d) level as shown in Figure 3.

[c] stability based on energetic order at B3LYP/cc-pVTZ level of theory.

Table S2. Results of DFT calculations on the smaller species.

$C_kH_lO_mS_n$

k	l	m	n	Formula	Strong bands in the fingerprint region [a]
2	2	2	1	c-CH=CH-OSO	1020, 1642
2	2	2	1	c-CH=CH-SOO	984, 1600
2	2	2	0	c-(H_2COC)O	916, 1953
2	2	2	0	HO-CC-OH	1197
2	2	2	0	c-CHO-CHO	1033, 1188
2	2	0	1	HCCSH(ethynethiol)	948
2	4	1	1	Cis-C_2H_3OSH	916, 1124, 1649
2	4	1	1	Cis-C_2H_3SOH	851, 1174
2	4	1	1	CH_3COSH	974, 1104, 1759
2	4	1	1	CH_3CSOH	1079, 1242, 1377
2	4	1	1	CHO-CH_2-SH	1260, 1767
2	4	1	1	CHS-CH_2-OH	1146, 1175, 1357

2	4	1	1	c-CH_2CH_2OS(1,2-oxathietane)	978
2	4	1	1	c-CH_2OCH_2S	1056
2	4	2	0	$HOCH_2CHO$	1097, 1409, 1746
2	4	2	0	c-CH_2CH_2OO(1,3-dioxetane)	915, 1046
2	4	2	0	(E)$HOCH_2CHOH$	883, 1112, 1377
2	4	2	0	(Z)$HOCH_2CHOH$	1082, 1217
1	2	1	1	HCOSH(thioformic acid)	933, 1747
1	2	1	1	HCSOH(1-thiaethyne-1-ol)	1208, 1436
1	2	1	1	HO-C=SH	1241
1	2	1	1	c-CH_2SO	1127
1	2	1	1	$CH_2=O^+-S^-$	1386, 1511
1	2	2	1	c-CH_2O_2S	1018
1	2	2	1	c-CH_2SO_2	998
1	2	2	1	$CH_2=SOO$	1173, 1292
1	4	1	1	CH_3SOH	1172
1	4	1	1	CH_3OSH	1016
1	4	1	1	$CH_3SH=O$	1098
1	4	1	1	$CH_2=SHOH$	992,1075
1	4	2	0	CH_3OOH	1337
1	4	2	1	$OHSOCH_3$(methanesulfinic acid)	1123, 1139
1	4	2	1	$CH_2=S(OH)_2$	1061, 1203
1	4	2	1	CH_3-S-O-OH	1299
1	4	2	1	CH_3-O-O-SH	
1	4	2	1	CH_3-O-S-OH	979
1	4	2	1	CH_3-SH-O_2	1302
1	4	2	1	CH_3-O-SH=O	984
2	4	0	1	C_2H_3-SH(vinylthiol)	954, 1618
0	2	1	1	$H_2S=O$	2218, 2231
0	2	2	1	$S(OH)_2$	1177
0	2	2	1	HS(=O)OH(sulfinic acid)	1132

[a] scaled wavenumber (factor 0.9603) from the results at B3LYP/6-31G(d) level of theory.
